# Lectures on Diseases of the Stomach and Intestines

**Published:** 1905-06

**Authors:** 


### Reviews of Books.

**Lectures on Diseases of the Stomach and Intestines.** By Boardman Reed, M.D. Pp. 1021. Bristol: John Wright & Co. 1905.

In this volume of lectures for practitioners and students the attempt has been made to give a plain and unpretentious clinical guide to the diagnosis and treatment of the stomach and intestines, with an account of their relations to other diseases, and of the most recent methods in diagnosis and treatment. The book includes also "the gastro-intestinal clinic," in which all such diseases are separately considered.

The contents are divided up into four sections, containing 82 chapters, and interspersed with 112 illustrations. Part I. is made up of three lectures giving the anatomic, physiologic, clinic, and diagnostic data. Part II. contains twelve chapters on the methods of examination. The author describes the various details of the physical examination of the patient, the method of outlining the stomach, the instruments used for the extraction of the stomach contents, and the test meals and preparations for testing the stomach contents. Chapters on the quantitative estimations and microscopic examinations of the stomach contents, the results of uranalysis, and the examination of fæces and blood follow.

Lecture XV. is a carefully prepared tabular statement, including the leading symptoms of gastro-intestinal affections, with a full summary of the diseases or other conditions which have been known to cause them. The following nineteen lectures contain a complete account of the prophylactic and general considerations concerning diet, dietotherapy and the various methods of treatment, medicinal, mechanical, electric, and hydro-therapeutic, which have been found to be more or less useful.

Part IV.—the gastro-intestinal clinic—is the major portion of the volume, consisting of forty-eight lectures. These chapters give an excellent resumé of our knowledge of the diseases of the stomach and intestines, and their medical treatment, and conclude with a concise synopsis of the modern advances of surgery in this field.

The volume is full of interesting, instructive, and often original observations; it is one for the busy practitioner, who will find it a reliable friend, whose author he may constantly consult, and whose experience cannot fail to be of much service. All the newer methods of diagnosis and treatment which conduce to accuracy and efficiency may here be learnt.

